# Using machine learning models to predict the duration of the recovery of COVID-19 patients hospitalized in Fangcang shelter hospital during the Omicron BA. 2.2 pandemic

**DOI:** 10.3389/fmed.2022.1001801

**Published:** 2022-11-02

**Authors:** Yu Xu, Wei Ye, Qiuyue Song, Linlin Shen, Yu Liu, Yuhang Guo, Gang Liu, Hongmei Wu, Xia Wang, Xiaorong Sun, Li Bai, Chunmei Luo, Tongquan Liao, Hao Chen, Caiping Song, Chunji Huang, Yazhou Wu, Zhi Xu

**Affiliations:** ^1^Respiratory and Critical Care Medical Center, Xinqiao Hospital, Army Medical University, Chongqing, China; ^2^Department of Health Statistics, Army Medical University, Chongqing, China; ^3^National Exhibition and Convention Center Fangcang Shelter Hospital, Shanghai, China; ^4^Department of Orthopedics, Xinqiao Hospital, Army Medical University, Chongqing, China; ^5^Department of Medical Administration, Xinqiao Hospital, Army Medical University, Chongqing, China; ^6^Academic Affairs Office, Army Medical University, Chongqing, China; ^7^Xinqiao Hospital, Army Medical University, Chongqing, China; ^8^Army Medical University, Chongqing, China

**Keywords:** COVID-19, omicron, Fangcang shelter, machine learning model, vaccination

## Abstract

**Background:**

Factors that may influence the recovery of patients with confirmed SARS-CoV-2 infection hospitalized in the Fangcang shelter were explored, and machine learning models were constructed to predict the duration of recovery during the Omicron BA. 2.2 pandemic.

**Methods:**

A retrospective study was conducted at Hongqiao National Exhibition and Convention Center Fangcang shelter (Shanghai, China) from April 9, 2022 to April 25, 2022. The demographics, clinical data, inoculation history, and recovery information of the 13,162 enrolled participants were collected. A multivariable logistic regression model was used to identify independent factors associated with 7-day recovery and 14-day recovery. Machine learning algorithms (DT, SVM, RF, DT/AdaBoost, AdaBoost, SMOTEENN/DT, SMOTEENN/SVM, SMOTEENN/RF, SMOTEENN+DT/AdaBoost, and SMOTEENN/AdaBoost) were used to build models for predicting 7-day and 14-day recovery.

**Results:**

Of the 13,162 patients in the study, the median duration of recovery was 8 days (interquartile range IQR, 6–10 d), 41.31% recovered within 7 days, and 94.83% recovered within 14 days. Univariate analysis showed that the administrative region, age, cough medicine, comorbidities, diabetes, coronary artery disease (CAD), hypertension, number of comorbidities, CT value of the ORF gene, CT value of the N gene, ratio of ORF/IC, and ratio of N/IC were associated with a duration of recovery within 7 days. Age, gender, vaccination dose, cough medicine, comorbidities, diabetes, CAD, hypertension, number of comorbidities, CT value of the ORF gene, CT value of the N gene, ratio of ORF/IC, and ratio of N/IC were related to a duration of recovery within 14 days. In the multivariable analysis, the receipt of two doses of the vaccination vs. unvaccinated (OR = 1.118, 95% CI = 1.003–1.248; *p* = 0.045), receipt of three doses of the vaccination vs. unvaccinated (OR = 1.114, 95% CI = 1.004–1.236; *p* = 0.043), diabetes (OR = 0.383, 95% CI = 0.194–0.749; *p* = 0.005), CAD (OR = 0.107, 95% CI = 0.016–0.421; *p* = 0.005), hypertension (OR = 0.371, 95% CI = 0.202–0.674; *p* = 0.001), and ratio of N/IC (OR = 3.686, 95% CI = 2.939–4.629; *p* < 0.001) were significantly and independently associated with a duration of recovery within 7 days. Gender (OR = 0.736, 95% CI = 0.63–0.861; *p* < 0.001), age (30–70) (OR = 0.738, 95% CI = 0.594–0.911; *p* < 0.001), age (>70) (OR = 0.38, 95% CI = 0292–0.494; *p* < 0.001), receipt of three doses of the vaccination vs. unvaccinated (OR = 1.391, 95% CI = 1.12–1.719; *p* = 0.0033), cough medicine (OR = 1.509, 95% CI = 1.075–2.19; *p* = 0.023), and symptoms (OR = 1.619, 95% CI = 1.306–2.028; *p* < 0.001) were significantly and independently associated with a duration of recovery within 14 days. The SMOTEEN/RF algorithm performed best, with an accuracy of 90.32%, sensitivity of 92.22%, specificity of 88.31%, F1 score of 90.71%, and AUC of 89.75% for the 7-day recovery prediction; and an accuracy of 93.81%, sensitivity of 93.40%, specificity of 93.81%, F1 score of 93.42%, and AUC of 93.53% for the 14-day recovery prediction.

**Conclusion:**

Age and vaccination dose were factors robustly associated with accelerated recovery both on day 7 and day 14 from the onset of disease during the Omicron BA. 2.2 wave. The results suggest that the SMOTEEN/RF-based model could be used to predict the probability of 7-day and 14-day recovery from the Omicron variant of SARS-CoV-2 infection for COVID-19 prevention and control policy in other regions or countries. This may also help to generate external validation for the model.

## Introduction

Since the first case of the Omicron variant of the SARS-CoV-2 infection was detected in Shanghai, China on March 1, 2022, the epidemic has spread rapidly, with the largest number of daily new confirmed cases reaching 5,487 on April 28 and asymptomatic infections reaching 25,173 on April 10. To maximize the protection of people's health and embody the concept of people first and life first, Fangcang shelter hospitals were quickly built and put into use in Shanghai. The National Exhibition Center Fangcang shelter hospital was set up with the capacity to accommodate up to 50,000 asymptomatic carriers and patients with mild pneumonia. The hospital has admitted and discharged more than 170,000 patients with asymptomatic and mild Omicron infections since April 9, 2022.

The dominant strain of the current epidemic wave was SARS-CoV-2 Omicron BA. 2.2, which represented a small sub-lineage of BA.2 worldwide seen in Hong Kong, the UK, and Australia previously. Omicron BA. 2.2 is more transmissible, but tends to be less virulent, with the majority of diseases observed in Shanghai being asymptomatic infection or mild illness. Despite this, the crude case fatality rate in people over 60 was 2.7% in Hong Kong (1). The dynamic model of SARS-CoV-2 transmission from the School of Public Health of Fudan University estimated that the Omicron epidemic spread in Shanghai would require 15.6 times the available capacity of the intensive care unit, and would cause −1.55 million deaths if the pandemic control strategies were lifted (2).

The pressure for the control of the omicron pandemic wave in China is still stressed until there is a comprehensive understanding of the clinical characteristics of the BA2.2 strain infections in the Chinese population. The duration of viral shedding is decisive in considering quarantine strategies and reducing transmission. For the original SARS-CoV-2, the medium duration of viral RNA shedding was 12 days after the onset of illness in hospitalized patients (3). In a recent study, researchers reported that the duration of viral RNA shedding for the Omicron variant was 7 days (IQR = 5–8 days) among fully vaccinated national football league players and staff members in the United States (3). However, there are few serial virus testing data available to analyze the viral clearance time for Omicron variants in the general population. Older age was reported to be a determinant of RNA persistence in nasopharyngeal samples (4). Treatment plans, including corticosteroid usage and Lopinavir/ritonavir administration, also led to prolonged viral RNA shedding (5). The Chinese government has enacted a COVID-19 vaccination program over the past 2 years, with booster shots for fully vaccinated people. However, whether vaccination is associated with the duration of virus clearance, specifically for the omicron variant, has not been reported.

In this study, we aim to describe factors that may influence the recovery of patients with confirmed SARS-CoV-2 Omincron BA. 2.2 infections hospitalized in the National Exhibition and Convention Center Fangcang hospital and to build machine learning models to predict the probability of recovery on day 7 and day 14 from the onset of disease during the Omicron BA. 2.2 wave. The findings provide a theoretical basis and reference for prevention and control strategies for the COVID-19 epidemic.

## Materials and methods

### Patients

Participants were retrospectively screened at the National Exhibition and Convention Center Fangcang shelter (Shanghai, China) from April 9, 2022 to April 25, 2022 during the Omicron BA. 2.2 strain pandemic period. This shelter is responsible for admitting confirmed COVID-19 patients classified as having an asymptomatic infection or mild illness based on the WHO COVID-19 treatment guidelines (6). The eligibility criteria required individuals to have a positive nucleic acid amplification test (NAAT) for COVID-19. The exclusion criteria included the following: (1) lack of key clinical data or inoculation information; and (2) moderate, severe, or critical illness. This study was approved by the Ethics Committee of Xinqiao Hospital (No. 2022-197-01). Informed consent was waived according to the retrospective nature of the study.

### Data collection and outcome

Medical documents were recorded using a WeChat Mini Program that enables patients' self-reported information to be assembled under the guidance of medical staff. Data including age, gender, living districts, occupation, oral medication, comorbidities, SARS-CoV-2-related symptoms (fever, headache, cough, sputum, malaise, stuffy/running nose, scratchy throat, muscle pain, nausea, vomiting, diarrhea, and loss of taste and smell), vaccination history, rounds of NAAT tests, and Ct values of the ORF lab gene, N gene, and internal control (IC) in every round were collected. The primary outcome was recovery on day 7 and day 14 from disease onset. Recovery was defined as being allowed to leave quarantine or hospital with two consecutive negative tests (at least 24 h apart) or Ct values ≥35 according to the 9th edition of COVID-19 diagnosis and treatment guidance issued by the Nation Health Commission of China (7).

### Statistics and model construction

Statistical analysis was performed using SPSS Statistics (IBM, version 26.0) and R (Version 4.1.1). Continuous variables were expressed as the median with IQR or mean ± SD, and categorical variables were expressed as the number and percent (%). Variables were compared using Fisher's exact tests, χ^2^ statistic, or Student's *t*-test. Variables with a *p*-value of <0.05 in the univariate test were included in the multivariable logistic regression model to screen out variables that had an independent impact on the recovery of Omicron infection on day 7 and day 14. Based on the variables screened using logistic regression, two consecutive negative rt-PCR results on 7-day or 14-day were used as outcome variables, and the dataset was randomly divided into a training set and test set in the ratio of 7:3. Predictive models were constructed to predict the 7-day and 14-day recovery rates of COVID-19 patients hospitalized in Fangcang shelter during the Omicron BA. 2.2 pandemic. Principal component analysis (PCA) was used to identify separate outcome groups with variables selected using logistic regression.

Python 3.7.6 was used to build machine learning predictive models. Five machine learning algorithms, that is, the decision tree (DT), support vector machine (SVM), random forest (RF), AdaBoost algorithm, and AdaBoost algorithm with DT as a classifier, were used. The outcomes of rehabilitation on day 7 and day 14 were used as dependent variables, and all the collected variables were included as independent variables. The dataset was divided into a training set, test set, and validation set in the ratio of 6:2:2. The training set was used to train different machine learning models and the test set was used to optimize the parameters of different machine learning models using a grid search method. Finally, the validation set was used to validate the efficacy of different machine learning models with optimal parameters. The prediction results were evaluated using the accepted accuracy, sensitivity, specificity, and F1 score, area under the curve (AUC), and Matthews correlation coefficient (MCC) as evaluation indicators. Finally, the SMOTEENN algorithm was used to balance the two datasets. The SMOTEENN algorithm is a combination of the SMOTE algorithm and the ENN algorithm. SMOTEENN is a balanced algorithm that combines oversampling and undersampling the dataset through the SMOTE algorithm and then uses the ENN algorithm to clean it. Batista et al. proposed it in 2004 (8). After the data were balanced, the prediction was made by the five aforementioned methods, and the prediction results before and after balancing were compared.

## Results

### Patients' characteristics

**Figure 1:**
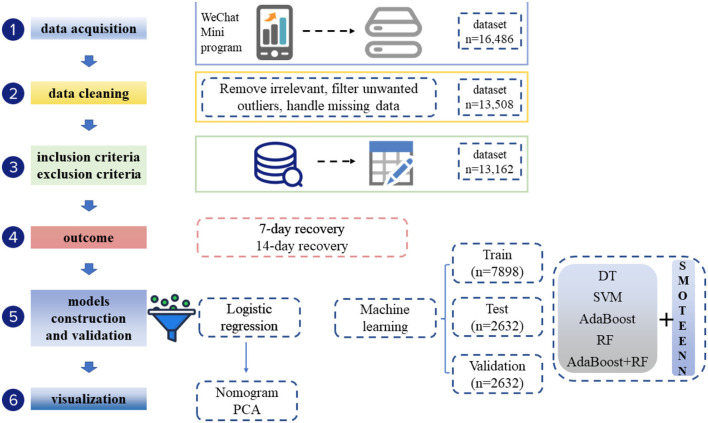
Flow chart of participant selection and the study design.

A total of 13,162 COVID-19 nucleic acid-positive cases from Hongqiao International Exhibition Center Fangcang shelter (Shanghai) were included (Figure 1). These patients came from 14 administrative regions of Shanghai. The geographic distribution of these Omicron-infected patients is visualized in Figure 1. Table 1 shows the details of the baseline characteristics of the enrolled patients. The median age of these patients was 43.0 (IQR, 31.0–53.0), 7,358 patients (57.27%) were male, and 12,575 patients (95.54%) and 587 (4.466%) patients were categorized as having asymptomatic and mild COVID-19, respectively. The common comorbidities included diabetes (357, 2.71%), CAD (20, 0.15%), hypertension (952, 7.23%), and cancer (7, 0.05%). The median CT value was 39.60 (IQR, 34.65–40.00) for the ORF gene and 38.59 (IQR, 33.19–40.00) for the N gene. The median ORF/IC ratio was 1.32 (IQR, 1.21–1.40) and median N/IC ratio was 1.30 (1.16–1.38). Among the patients, 11,167 cases (84.84%) had a history of SARS-CoV-2 vaccination, 6,324 (48.05%) participants received three doses, 4,352 (33.06%) cases received two doses, and 491 (3.73%) received one dose.

**Table 1:** Clinical characteristics of 13,162 cases of Omicron infection in Shanghai.

**Variables**		**Variables**	
**Sex**		N	12,956 (98.43)
Male	7,538 (57.27)	Y	206 (1.57)
Female	5,624 (42.73)	**Cold medicine**	
**Age**		N	12,887 (97.91)
**< 30**	3,068 (23.31%)	Y	275 (2.09)
30-70	8,710 (66.18%)	**Comorbidities**	
>70	1,384 (10.51%)	N	11,964 (90.90)
**Marriage**		Y	1,198 (9.10)
Married	12,746 (96.84)	**Diabetes**	
Single	389 (2.96)	N	12,805 (97.29)
Divorced	27 (0.21)	Y	357 (2.71)
**Diagnosis**		**Coronary artery disease**	13,142 (99.85)
Asymptomatic infection	12,575 (95.54)	N	20 (0.15)
Mild illness	587 (4.46)	Y	
**Vaccination dose**		**Hypertension**	
0 None	1,995 (15.16)	N	12,210 (92.77)
1 dose	491 (3.73)	Y	952 (7.23)
2 doses	4,352 (33.06)	**Cancer**	
3 doses	6,324 (48.05)	N	13,155 (99.95)
**Chinese medicine prescription**		Y	7(0.05)
N	10,100 (76.74)	**Other comorbidities**	
Y	3,062 (23.26)	N	13,149 (99.90)
		Y	13(0.10)
**Lianhua Qingwen**		**Number of comorbidities**	
N	10,548 (80.14)	0	11,964 (90.90)
Y	2,614 (19.86)	1	1,017 (7.73)
**Kangbingdu Granules**		2	181 (1.38)
N	13,095 (99.49)	**Fever**	
Y	67 (0.51)	N	12,925 (98.20)
**Other Chinese medicine prescription**		Y	237 (1.80)
N	12,738 (96.78)	**CT value of ORF gene**	39.60 [34.65- 40.00]
Y	424 (3.22)	**CT value of N gene**	38.59 [33.19, 40.00]
**Cough medicine**		**ORF/IC**	1.32 [1.21, 1.40]
N	12,180 (92.54)	**N/IC**	1.30 [1.16, 1.38]
Y	982 (7.46)		
**Antipyretics**			

### Identifying prognostic factors associated with 7-day recovery and 14-day recovery

**Figure 2:**
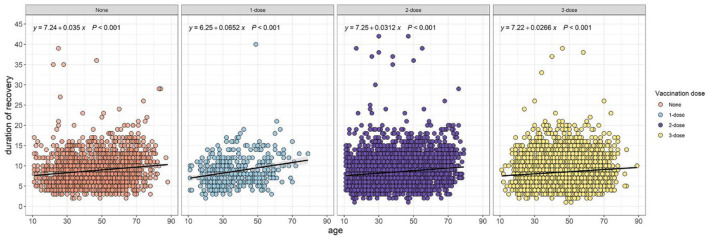
Correlation between age and recovery duration assessed using Spearman correlation.

The univariate analysis results revealed the potential prognostic factors associated with 7-day and 14-day recovery, and are presented in Table 1. The analysis demonstrated that the administrative region, age, cough medicine, comorbidities, diabetes, CAD, hypertension, number of comorbidities, CT value of the ORF gene, CT value of the N gene, ratio of ORF/IC, and ratio of N/IC were potential prognostic factors that affected the 7-day recovery of COVID-19 patients. Data regarding age, gender, vaccination dose, cough medicine, comorbidities, diabetes, CAD, hypertension, number of comorbidities, CT value of the ORF gene, CT value of the N gene, ratio of ORF/IC, and ratio of N/IC were potential prognostic factors that affected 14-day recovery. The relationship between the duration of recovery and age was performed using Spearman correlation stratified by the vaccination dose. Regardless of the vaccination status, a positive correlation was observed in unvaccinated (*p* < 0.001), one-dose (*p* < 0.001), two-dose (*p* < 0.001) and three-dose (*p* < 0.001) participants (Figure 2).

**Figure 3:**
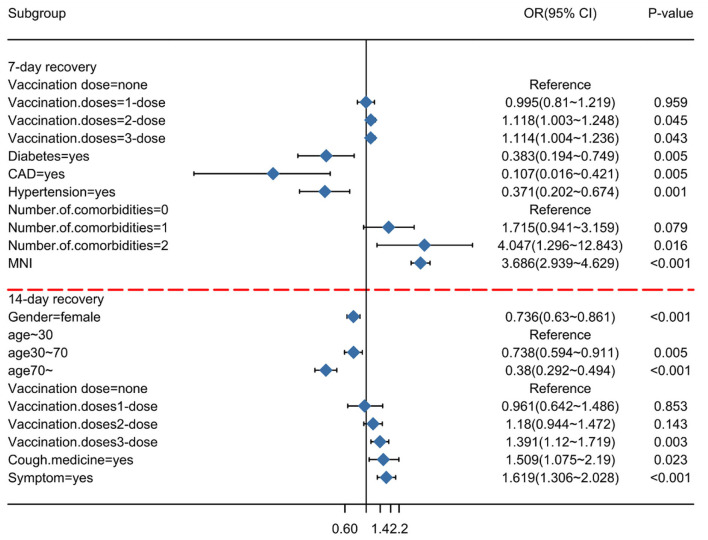
Subgroup analysis of Omicron infection recovery. An odds ratio of <1 implies a lower risk of recovery on the indicated day.

To minimize the effect of confounders, these prognostic factors were subsequently included in the multivariate logistic regression analysis (Table 2). In the multivariable analysis, the receipt of two doses of the vaccination vs. unvaccinated (OR = 1.118, 95% CI = 1.003–1.248; *p* = 0.045), receipt of three doses of the vaccination vs. unvaccinated (OR = 1.114, 95% CI = 1.004–1.236; *p* = 0.043), diabetes (OR = 0.383, 95% CI = 0.194–0.749; *p* = 0.005), CAD (OR = 0.107, 95% CI = 0.016–0.421; *p* = 0.005), hypertension (OR = 0.371, 95% CI = 0.202–0.674; *p* = 0.001), and the ratio of N/IC (OR = 3.686, 95% CI = 2.939–4.629; *p* < 0.001) were significantly and independently associated with a duration of recovery within 7 days. Gender (OR = 0.736, 95% CI = 0.63–0.861; *p* < 0.001), age (30–70) (OR = 0.738, 95% CI = 0.594–0.911; *p* < 0.001), age (>70) (OR = 0.38, 95% CI = 0292–0.494; *p* < 0.001), receipt of three doses of the vaccination vs. unvaccinated (OR = 1.391, 95% CI = 1.12–1.719; *p* = 0.0033), cough medicine (OR = 1.509, 95% CI = 1.075–2.19; *p* = 0.023), and symptoms (OR = 1.619, 95% CI = 1.306–2.028; *p* < 0.001) were significantly and independently associated with a duration of recovery within 14 days. A forest plot was used to visualize the results of the multivariate logistic regression analysis (Figure 3).

**Table 2:** Multivariable logistic regression models for Omicron infection recovery.

**Variable**	**7-day recovery**	***P*-Value**	**14-day recovery**	***P*-Value**
	**OR (95%CI)**		**OR (95%CI)**	
Vaccination dose = none	Reference		Reference	
Vaccination doses = 1-dose	0.995 (0.81–1.219)	0.959	0.961 (0.642–1.486)	0.853
Vaccination doses = 2-dose	1.118 (1.003–1.248)	0.045	1.18 (0.944–1.472)	0.143
Vaccination doses = 3-dose	1.114 (1.004–1.236)	0.043	1.391 (1.12–1.719)	0.003
Diabetes = yes	0.383 (0.194–0.749)	0.005	–	–
CAD = yes	0.107 (0.016–0.421)	0.005	–	–
Hypertension = yes	0.371 (0.202–0.674)	0.001	–	–
Number of comorbidities = 0	Reference		–	–
Number of comorbidities = 1	1.715 (0.941–3.159)	0.079	–	–
Number of comorbidities = 2	4.047 (1.296–12.843)	0.016	–	–
MNI	3.686 (2.939–4.629)	< 0.001	–	–
Gender = female			0.736 (0.63–0.861)	< 0.001
age−30			Reference	
Age 30–70			0.738 (0.594–0.911)	0.005
Age 70–			0.38 (0.292–0.494)	< 0.001
Cough medicine = yes			1.509 (1.075–2.19)	0.023
Symptom = yes			1.619 (1.306–2.028)	< 0.001

### Logistic regression model and PCA

According to the variables screened above, first, a logistic regression model was constructed to predict recovery on day 7, with variables including vaccination doses, diabetes, CAD, hypertension, number of comorbidities, and ratio of N/IC. However, the predictive value of the model was not acceptable, and had an AUC of 56.8% for the test set. Similarly, when variables including vaccination dose, gender, age, cough medicine, and symptoms were used to construct the 14-day predictive model, this yielded an AUC of 63.01% for the test set. Then PCA showed there was a large degree of overlap between the two groups for the 7-day and 14-day recovery outcomes based on the selected variables in the logistic regression. Some sample points were scattered in other areas, which indicates that there were discrete recovered patients and isolated patients or outliers (Figure 2).

### Machine learning models

To build a more robust predictive model, five machine learning-based prediction models were established (DT, RF, SVM, AdaBoost, and DT+AdaBoost) for 7-day recovery and 14-day recovery from Omicron infection. Table 3 shows the accuracy, sensitivity, specificity, F-measure, AUC, and MCC of each model evaluated on the validation set. The five models performed poorly, with AUC <60% for both 7-day and 14-day recovery models based on the original dataset.

**Table 3:** Evaluation indices of the classification models for full variables.

**Model**	**7-day**						**14-day**					
	**Sen**	**Sp**	**Acc**	***F*-measure**	**MCC**	**AUC**	**Sen**	**Sp**	**Acc**	***F*-measure**	**MCC**	**AUC**
DT	0.2973	0.8291	0.6092	0.3849	0.1468	0.5622	1.0000	0.0000	0.9502	0.9745	0.0000	0.5000
RF	0.4109	0.7374	0.6031	0.4599	0.1560	0.5742	0.9956	0.0229	0.9472	0.9729	0.0553	0.5093
SVM	0.2188	0.9026	0.6213	0.3222	0.1685	0.5607	1.0000	0.0000	0.9502	0.9745	0.0000	0.5000
AdaBoost	0.3564	0.8110	0.6240	0.4381	0.1883	0.5837	1.0000	0.0000	0.9502	0.9745	0.0000	0.5000
DT+AdaBoost	0.3694	0.7793	0.6103	0.4375	0.1600	0.5734	0.9980	0.0076	0.94873	0.9737	0.0257	0.5028
DT+SMOTEENN	0.8689	0.8481	0.8588	0.8631	0.7173	0.8585	0.7248	0.8895	0.8095	0.7871	0.6246	0.8072
RF+SMOTEENN	0.9222	0.8831	0.9032	0.9071	0.8066	0.8975	0.9340	0.9381	0.9361	0.9342	0.8721	0.9353
SVM+SMOTEENN	0.8311	0.6098	0.7232	0.7548	0.4532	0.7205	0.5461	0.8479	0.7013	0.6399	0.4147	0.6970
AdaBoost+SMOTEENN	0.7644	0.7103	0.7380	0.7495	0.4756	0.7374	0.6038	0.6181	0.6112	0.6014	0.2218	0.6110
DT+AdaBoost+SMOTEENN	0.8489	0.8294	0.8394	0.8442	0.6972	0.8392	0.8166	0.9589	0.8897	0.8780	0.7881	0.8877

**Figure 4:**
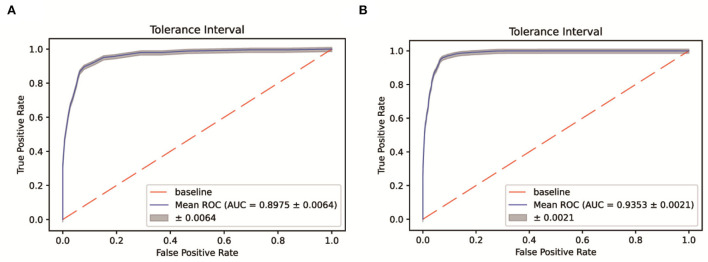
ROC-AUC of the 7-day **(A)** and 14-day **(B)** recovery models based on the SMOTEENN/RF algorithm.

Patients were sampled from a single Fangcang shelter. The selection bias and data imbalance contributed to the low accuracy of the 7-day recovery models and falsely high accuracy of the 14-day recovery models for the above algorithms. Therefore, the SMOTEENN algorithm was used to perform the balance process for the original dataset. The balanced sample size of the recovered vs. unrecovered cohort was 835 vs. 1,996 for the 7-day recovery dataset, and 10,599 vs. 435 for the 14-day recovery dataset. Then five machine learning algorithms were applied to develop prediction models based on the balanced dataset. The SMOTEENN+RF algorithm achieved the best prediction results for both the 7-day and 14-day recovery models, that is, AUCs of 89.75% and 93.53% for the validation set, respectively (Figure 4).

## Discussion

During the Omicron BA. 2.2 pandemic wave in Shanghai, over 100 Fangcang shelter hospitals were developed and served to isolate patients with mild to moderate COVID-19. However, no detailed published reports have investigated the demographic and clinical information, viral load shedding time, or factors that influenced the recovery of patients with confirmed SARS-CoV-2 infection hospitalized in the Fangcang shelter.

In the present study, 13,162 COVID-19 patients composed of asymptomatic carriers and mild cases were enrolled from the National Exhibition Center Fangcang shelter in Shanghai. The collected data, including age, gender, comorbidities, vital signs, therapeutic drugs, results of nucleic acid detection, and viral load shedding time, were analyzed to identify risk factors that may influence SARS-CoV-2 RNA shedding. The results showed that the median duration of disease recovery was 8 days (IQR 6–10 d). At 7 days, the rate of recovery from omicron infection was 41.31%. Vaccination, diabetes, CAD, hypertension, ratio of N/IC were independent factors associated with the 7-day recovery of patients with confirmed SARS-CoV-2 infection hospitalized at the Fangcang shelter. At 14 days, the rate of recovery was 94.83%. Gender, age, three doses of the vaccination, cough medicine, and symptoms were observed to be independently associated with 14-day recovery. Machine learning models were constructed to predict the recovery of COVID-19 on day 7 and day 14.

The Omicron variant was first found in South Africa at the end of November 2021. As of May 2022, the Omicron variant of concern became the dominant variant circulating globally, and evolved into many distinct sub-lineages: BA.1, BA.2, BA.3, BA.4, and BA.5 (9). According to WHO epidemiological update reports, BA.2 and its descendent lineages (i.e., BA.2.X) were the dominant variants from the 1st week of May 2022, comprising 97% of all sequences submitted to GISAID. The prevalence of BA.1 and its descendent lineages (i.e., BA.1.X), BA.3, and the B.1.617.2 (Delta) variant significantly decreased, falling below a global prevalence of <1% (9).

In several studies, researchers reported that Omicron variants had 70-fold higher replication competence in the human bronchus, but low viral replication in the lungs compared with the original COVID-19 strain or Delta strain 24 h post-infection (10). The BA.2 variant, with eight unique spike alterations compared with the BA.1 variant (11), has a higher effective reproduction number, higher fusogenicity, and higher pathogenic potential than the BA.1 variant (12). This evidence reasoned the rapid spread of Omicron BA. 2.2 in Shanghai with most infected victims being asymptomatic or having mild illness. In our study, 95.59% of the included patients had asymptomatic infections (12,911/13,508). Of the remaining 597 cases with mild illness, only 240 cases presented with fever.

As reported by Maslo et al., a significantly decreased demand in oxygen therapy was observed during the Omicron wave compared with the oxygen need during the Delta wave in South Africa (17.6 vs. 74%; *P* < 0.001); this was also the case for the percentage of mechanical ventilation requirement (1.6 vs. 12.4%; *P* < 0.001) (13). Maslo et al. suggested that the decreased severity of Omicron infection may be associated with vaccination. Now, this new sub-lineage of BA.2 is causing mayhem in many countries, such as the UK, Australia, and China (14). Previously, the outbreak of Omicron BA. 2.2 in Hong Kong claimed 6,356 lives, and 90% of these deceased people had not received the COVID-19 vaccination (15).

In many studies, researchers have shown that Omicron variants have substantial immunologic escape ability from neutralizing antibodies induced by vaccination (16–19). However, a booster dose of mRNA vaccines, either Moderna or Pfizer, can still induct consistent neutralizing antibody titers against either BA.1 or BA.2 (19, 20). In a recent study, researchers also suggested that promoting the booster shot is an effective means to prevent the transmission of SARS-CoV-2, particularly to withstand the transmission of the Omicron strain. Moreover, a retrospective study in which 23,391 COVID-19 cases and 46,64 controls from a nationwide pharmacy-based testing program in the US were analyzed and showed that the prior receipt of three mRNA vaccine doses prevented the development of symptomatic SARS-CoV-2 for both Omicron and Delta. In the present study, it was first confirmed that two doses of SARS-CoV-2 vaccine (BBIBP-CorV or CoronaVac) increased the probability of SARS-CoV-2 nucleic acid negative conversion within 14 days after infection. Three doses of CoronaVac or a booster dose of BBIBP-CorV shortened the duration of virus clearance within 7 days and 14 days after infection. Hence, it makes sense to devise different vaccination rollouts optimally (21).

In the present study, the median duration of RNA shedding was 8 days, which is shorter than the 2 weeks of RNA shedding of the original SARS-CoV-2 strain reported previously (4). Nationwide vaccination may contribute to the accelerated viral clearance of the Omicron BA. 2.2 wave. It was noticed that age is associated with prolonged viral RNA shedding, which is consistent with a previous report (4). This can also be attributed to the age-dependent impairment of innate and adaptive immunity, which could make it more difficult for older patients to eradicate pathogens. In contrast to earlier findings, however, in this study, the results showed that female patients tended to have prolonged RNA shedding compared with male patients at day 14, but tended to become negative at day 7 compared with male patients. This contradictory finding may involve an underlying mechanism for sex-related differences in SARS-CoV-2 clearance related to the presence of sex hormones, which influence different components of the immune system.

Understanding the recovery of COVID-19 during Omicron BA. 2.2 is of great importance in terms of enabling the government to set up a quarantine and lockdown policy to inform public health guidance. Fangcang shelter hospitals provide isolation for asymptomatic infection or mild illness, and are characterized by rapid construction, massive scale, and low cost (2). In the present study, it was noticed that cough medicine increased the probability of recovery on day 7, which reinforces the importance of oral prescription during quarantine in Fangcang shelter Hospitals. At the summit of the Shanghai pandemic, the average number of new cases was >20,000 per day, and the timely turnover of Fangcang shelter Hospitals was important for preventing panic caused by medical shortages. Using the 7-day and 14-day recovery machine learning-based predictive model (SMOTEENN/RF), the recovery of patients was predicted and it was possible to prepare for available beds within the subsequent 1 or 2 weeks. Machine learning-based models have been used in the screening, diagnosis, severity assessment, prognosis, epidemiology, and even dietary guidance of COVID-19 (21–26) (Table 2). In the present study, a machine learning-based model was used to predict the recovery of Omicron BA. 2.2 infected COVID-19 patients, and the results can be used as a reference to guide quarantine or isolation policy.

The study has several limitations. First, although it has been reported that the virus strains detected in Shanghai were all Omicron BA. 2.2, personalized genotyping of the virus was not available. Second, information about the vaccination type was not accessible because clinical data were collected using a self-report WeChat Mini Program. Third, external validation is required to test the accuracy of the proposed models.

## Data availability statement

The original contributions presented in the study are included in the article/Supplementary material, further inquiries can be directed to the corresponding author/s.

## Ethics statement

The studies involving human participants were reviewed and approved by Medical Ethics Committee of the Second Affiliated Hospital of the PLA Army Medical University. Written informed consent to participate in this study was provided by the participants' legal guardian/next of kin.

## Author contributions

ZX is the guarantor of the manuscript. YX wrote the manuscript and generated the figures and tables. WY and QS researched the data. LS reviewed/edited the manuscript before submission. YW managed the result analysis. TL, HC, CS, and CH contributed to a discussion on the content and writing of the manuscript. All authors contributed to the article and approved the submitted version.

## Funding

This work was supported by the Key Support Object Training Project of Army Medical University (Third Military Medical University) (No. 2019R025) and the National Natural Science Foundation of China (No. 82173621).

## Conflict of interest

The authors declare that the research was conducted in the absence of any commercial or financial relationships that could be construed as a potential conflict of interest.

## Publisher's note

All claims expressed in this article are solely those of the authors and do not necessarily represent those of their affiliated organizations, or those of the publisher, the editors and the reviewers. Any product that may be evaluated in this article, or claim that may be made by its manufacturer, is not guaranteed or endorsed by the publisher.
