# Supplementary material for: Using machine learning models to predict the duration of the recovery of COVID-19 patients hospitalized in Fangcang shelter hospital during the Omicron BA. 2.2 pandemic
Source: Front Med (Lausanne). 2022 Nov 2;9:1001801. doi: 10.3389/fmed.2022.1001801 (PMC9666500; doi:10.3389/fmed.2022.1001801)
Supplement: Supplementary file 1 [file Data_Sheet_1.PDF]

Supplementary Figures and Tables

Supplementary Figure 1. The geographic distribution of these Omicron-infected patients.

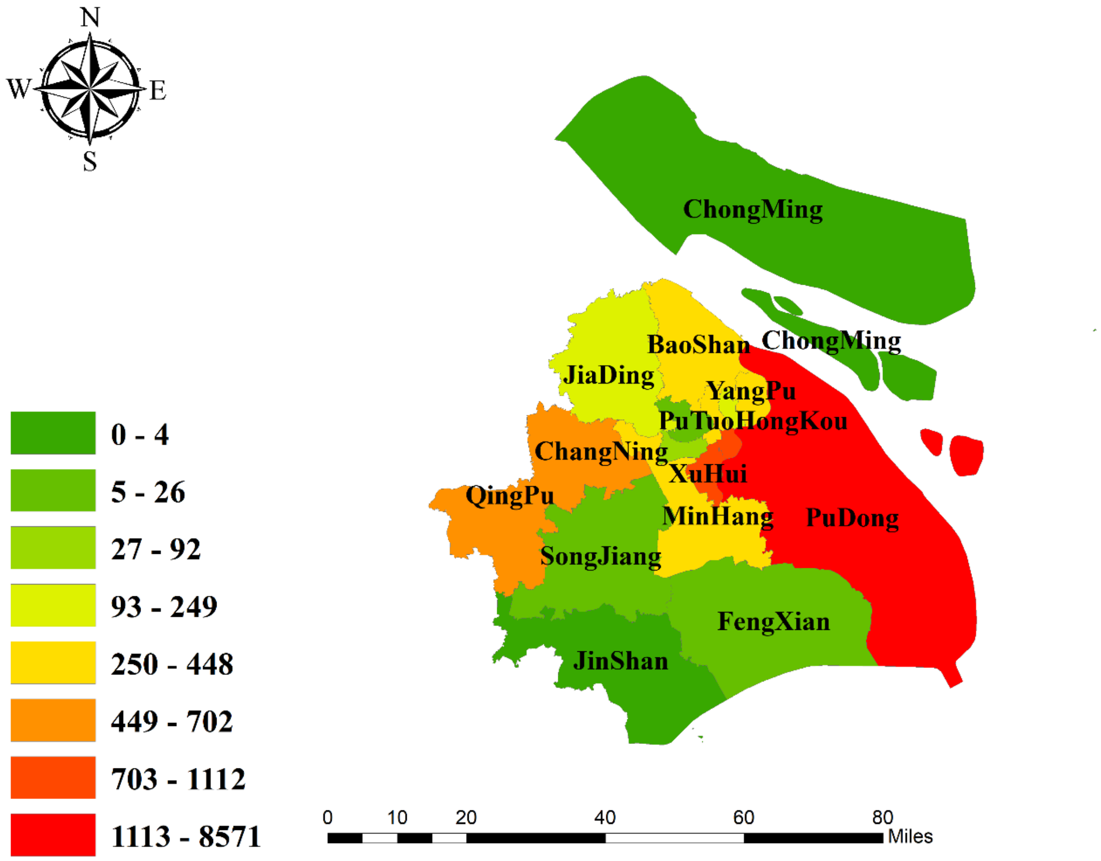

Supplementary Figure 2. Principal component analysis of 7-day recovery dataset (A) and 14-day recovery dataset (B).3D

A B

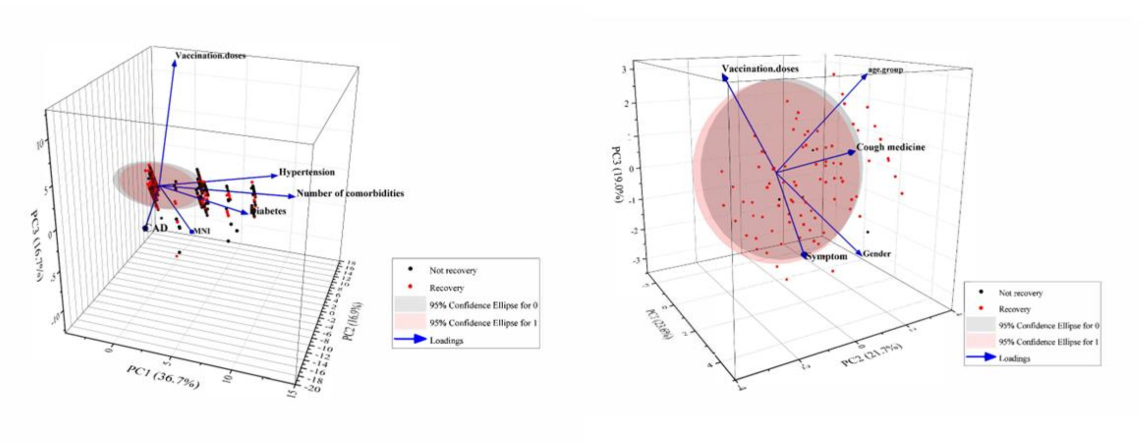

Supplementary Table 1. Univariate analysis of Omicron infection recovery.

variables	7-day		p	14-day		p
	No.of Infection(%)	No.of Recovery(%)		No.of Infection(%)	No.of Recovery(%)	
n	7725	5437		680	12482	
Region = Pudong	4999 (64.71)	3306 (60.81)	<0.001	433 (63.68)	7872 (63.07)	0.779
Gender = female	3286 (42.54)	2338 (43.00)	0.608	340 (50.00)	5284 (42.33)	<0.001
Age (median [IQR])	44.00 [32.00, 54.00]	40.00 [30.00, 52.00]	<0.001	47.00 [34.00, 58.00]	42.00 [31.00, 53.00]	<0.001
Marrige			0.891			0.462
Married	7485 (96.89)	5261 (96.76)		661 (97.21)	12085 (96.82)	
Single	225 (2.91)	164 (3.02)		19 (2.79)	370 (2.96)	
Divorced	15 (0.19)	12 (0.22)		0 (0.00)	27 (0.22)	
age.group			<0.001			<0.001
~30	1584 (20.50)	1484 (27.29)		115 (16.91)	2953 (23.66)	
30~70	5163 (66.83)	3547 (65.24)		428 (62.94)	8282 (66.35)	
70~	978 (12.66)	406 (7.47)		137 (20.15)	1247 (9.99)	
duration.recovery (median [IQR])	10.00 [8.00, 12.00]	6.00 [5.00, 7.00]	<0.001	16.00 [15.00, 17.25]	8.00 [6.00, 10.00]	<0.001
duration.hospital (median [IQR])	7.00 [5.00, 8.00]	6.00 [5.00, 7.00]	<0.001	6.00 [5.00, 7.00]	6.00 [5.00, 7.00]	0.953
Diagnosis = 2	354 (4.58)	233 (4.29)	0.441	25 (3.68)	562 (4.50)	0.357
Vaccination.doses			0.051			<0.001
None	1220 (15.79)	775 (14.25)		141 (20.74)	1854 (14.85)	
1-dose	300 (3.88)	191 (3.51)		29 (4.26)	462 (3.70)	

2-dose	2519 (32.61)	1833 (33.71)		226 (33.24)	4126 (33.06)	
3-dose	3686 (47.72)	2638 (48.52)		284 (41.76)	6040 (48.39)	
Chinese.medicine.prescription = yes	1835 (23.75)	1227 (22.57)	0.118	156 (22.94)	2906 (23.28)	0.874
Lianhua.Qingwen = yes	1567 (20.28)	1047 (19.26)	0.152	128 (18.82)	2486 (19.92)	0.518
Kangbingdu.Granules = yes	38 (0.49)	29 (0.53)	0.838	2 (0.29)	65 (0.52)	0.595
Other.Chinese.medicine = yes	255 (3.30)	169 (3.11)	0.571	29 (4.26)	395 (3.16)	0.141
Types.of.Chinese.medicine			0.265			0.975
0	5890 (76.25)	4210 (77.43)		524 (77.06)	9576 (76.72)	
1	1811 (23.44)	1209 (22.24)		154 (22.65)	2866 (22.96)	
2	24 (0.31)	18 (0.33)		2 (0.29)	40 (0.32)	
Cough.medicine = yes	620 (8.03)	362 (6.66)	0.004	34 (5.00)	948 (7.59)	0.015
Antipyretics = yes	124 (1.61)	82 (1.51)	0.711	8 (1.18)	198 (1.59)	0.497
Cold.medicine = yes	155 (2.01)	120 (2.21)	0.465	12 (1.76)	263 (2.11)	0.638
Comorbidities = yes	810 (10.49)	388 (7.14)	<0.001	92 (13.53)	1106 (8.86)	<0.001
Diabetes = yes	240 (3.11)	117 (2.15)	0.001	31 (4.56)	326 (2.61)	0.003
CAD = yes	18 (0.23)	2 (0.04)	0.009	3 (0.44)	17 (0.14)	0.138
Hypertension = yes	651 (8.43)	301 (5.54)	<0.001	73 (10.74)	879 (7.04)	<0.001
Cancer = yes	2 (0.03)	5 (0.09)	0.217	0 (0.00)	7 (0.06)	1.000
Other.comorbidities = yes	5 (0.06)	8 (0.15)	0.230	0 (0.00)	13 (0.10)	0.830
Number.of.comorbidities			<0.001			<0.001
0	6915 (89.51)	5049 (92.86)		588 (86.47)	11376 (91.14)	
1	689 (8.92)	328 (6.03)		76 (11.18)	941 (7.54)	

2	121 (1.57)	60 (1.10)		16 (2.35)	165 (1.32)	
Fever = yes	149 (1.93)	88 (1.62)	0.211	15 (2.21)	222 (1.78)	0.504
Symptom = yes	1696 (21.95)	1204 (22.14)	0.812	99 (14.56)	2801 (22.44)	<0.001
MO (median [IQR])	38.81 [34.03, 40.00]	40.00 [36.43, 40.00]	<0.001	40.00 [35.38, 40.00]	39.56 [34.61, 40.00]	0.005
MN (median [IQR])	37.63 [32.54, 40.00]	39.20 [35.10, 40.00]	<0.001	39.14 [33.67, 40.00]	38.57 [33.17, 40.00]	0.013
MOI (median [IQR])	1.31 [1.19, 1.39]	1.33 [1.24, 1.40]	<0.001	1.34 [1.24, 1.41]	1.32 [1.20, 1.40]	<0.001
MNI (median [IQR])	1.28 [1.14, 1.38]	1.32 [1.20, 1.39]	<0.001	1.32 [1.18, 1.40]	1.30 [1.16, 1.38]	0.003

STable 2

Comparison of updated studies using machine learning-based models for COVID-19 pandemic

Study	Country/Region	Year	Number of patients	Virus strain	Objective	Variables	Methods	Evaluation metric	Data Source	Potential application
ElAraby et al.[1]	NA	2021	NA	Not specified	Optimization Covid-19 screening with CXR	CXR image	Deep Learning + Crawler	Accuracy = 95.6% (for two-class labels) and 92.67% (for three-class labels)	MOMA dataset (603 chest X-ray images which were composed of 221 COVID-19, 382 non-COVID images and data from Kaggle and Github (455 COVID-19/ Normal)	The proposed crawler architecture is implemented using Java language concluded IDE development based on Oxygen and JDK-8. They utilized a central database that is designed to crawl the extracted Uniform Resource Locators URLs from different distributed web pages.
Shams et al.[2]	NA	2021	NA	Not specified	Foods and COVID-19 related death	Features in the database	Machine learning regression model (ridge regression, simple linear regularization, and elastic net	For Elastic Net Regression, the Mean Square Error (MSE) = 0.00018113, Root Mean Square Error (RMSE) = 0.01345867, Mean Absolute Error (MAE) = 0.00873109, and R2 metrics=0.09001016, and 20-	the COVID-19 Healthy Diet Dataset https://www.kaggle.com/mariaren	The results of this study may direct patients to eat particular types of food to reduce the possibility of becoming infected with the COVID-19 virus

							regression), and AdaBoost models.	fold cross-validation; Accuracy = 98.76%	/covid19-healthy-diet-dataset.	
Parino et al.[3]	Italy	2021	NA	Not specified	two-dose vaccination rollout plan	Epidemiological data and hypothetical vaccination plan	an extension of a discrete-time, deterministic SIR population model	NA	https://gitlab.com/PolitioComplexSystemLab/a-model-predictive-control-approach-to-optimally-Design-a-a-two-shot-Vanction-Rollow	to assist public health authorities in optimally planning the vaccination rollout
Park et al.[4]	Korea	2022	149,471	Not specified	severity assessment	patients' basic personal data (without vaccination information)	a tree-based gradient boosting machine learning model with binary logistic objectives, XGBoost (XGB)	For XGBoost model, area under the curve of receiver operating characteristic (AUROC) = 0.950, area under the precision-recall curve (AUPRC) = 0.268, F1 score = 0.861, precision = 0.923, sensitivity (Youden's index = 0.739), and specificity = 0.933.	a data set collected from February 2020 to July 2021 by the Korea Disease Control and Prevention Agency (KDCA).	This prediction model, trained with patient-generated health data (PGHD) from nationwide COVID-19 screening centers, can be globally utilized to monitor hospitalized or quarantined patients with confirmed SARS-CoV-2 infection daily

Amaral et al.[5]	São Paulo, Brazil	2021	NA	Not specified	predicting Infections, Recoveries, Deaths, and Viral Reproduction Numbers	Epidemiological data including number of infected, recovered, or death COVID-19 cases	A Time-Dependent SIR-Based Model	Mean Absolute Percentage Error (MAPE), which were less than 1 in almost all the measurements.	www.spcovid.net.brhttps://github.com/CSSEGISandData/COVID-19 https://covid.saude.gov.br	To guide government actions mainly in two basic aspects: real-time data assessment and dynamic predictions of Covid-19 curves for different regions of the state
VERDE et al. [6]	NA	2021	1,027	Not specified	Covid-19 screening	Coughing, breathing and voice sounds, in addition to data relating to health status, gender, age, certain pre-existing health conditions	Waikato Environment for Knowledge Analysis project (WEKA)	For SVM distinguishes healthy and pathological subjects, Accuracy = 97.07%, F1-score = 82.35%, specificity = 97.37%, precision = 73.68%, recall = 93.33%, AUC = 0.954	the Coswara database https://github.com/iiscleap/Coswara-Data	To supporting the early detection of the Covid-19 disorde
**Ours**	Shanghai, China	2022	13,162	Omicron BA. 2.2	Predicting recovery of infected individual	patients' demographic, clinical data, and inoculation history	RF+SMOTEENN	For 7-day recovery prediction, accuracy = 90.32%, sensitivity = 92.22% , specificity = 88.31%, F-measure = 90.71%, AUC = 90.27%, MCC=0.8066 ;For 14-day recovery prediction, accuracy	A dataset collected by a self-report WeChat Mini Program (non-public database)	To predict the probability of 7-day, 14-day recovery from the Omicron BA. 2.2 Variant of SARS-CoV-2 infection for the COVID-19 prevention and control policy in other regions or countries.

								= 93.81%, sensitivity = 93.40% , specificity = 93.80%, F-measure = 93.42%, AUC = 93.60%, MCC=0.8721		

References

1. ElAraby, M.E., et al., *A novel Gray-Scale spatial exploitation learning Net for COVID-19 by crawling Internet resources.* Biomed Signal Process Control, 2022. **73**: p. 103441.
2. Shams, M.Y., et al., *HANA: A Healthy Artificial Nutrition Analysis model during COVID-19 pandemic.* Comput Biol Med, 2021. **135**: p. 104606.
3. Parino, F., et al., *A model predictive control approach to optimally devise a two-dose vaccination rollout: A case study on COVID-19 in Italy.* Int J Robust Nonlinear Control, 2021.
4. Park, M.S., et al., *Machine Learning-Based COVID-19 Patients Triage Algorithm Using Patient-Generated Health Data from Nationwide Multicenter Database.* Infect Dis Ther, 2022. **11**(2): p. 787-805.
5. Amaral, F., et al., *Towards Providing Effective Data-Driven Responses to Predict the Covid-19 in Sao Paulo and Brazil.* Sensors (Basel), 2021. **21**(2).
6. Verde, L., et al., *Exploring the Use of Artificial Intelligence Techniques to Detect the Presence of Coronavirus Covid-19 Through Speech and Voice Analysis.* IEEE Access, 2021. **9**: p. 65750-65757.
